# Modeling Exon-Specific Bias Distribution Improves the Analysis of RNA-Seq Data

**DOI:** 10.1371/journal.pone.0140032

**Published:** 2015-10-08

**Authors:** Xuejun Liu, Li Zhang, Songcan Chen

**Affiliations:** College of Computer Science and Technology, Nanjing University of Aeronautics and Astronautics, Nanjing, China; Mayo Clinic, UNITED STATES

## Abstract

RNA-seq technology has become an important tool for quantifying the gene and transcript expression in transcriptome study. The two major difficulties for the gene and transcript expression quantification are the read mapping ambiguity and the overdispersion of the read distribution along reference sequence. Many approaches have been proposed to deal with these difficulties. A number of existing methods use Poisson distribution to model the read counts and this easily splits the counts into the contributions from multiple transcripts. Meanwhile, various solutions were put forward to account for the overdispersion in the Poisson models. By checking the similarities among the variation patterns of read counts for individual genes, we found that the count variation is exon-specific and has the conserved pattern across the samples for each individual gene. We introduce Gamma-distributed latent variables to model the read sequencing preference for each exon. These variables are embedded to the rate parameter of a Poisson model to account for the overdispersion of read distribution. The model is tractable since the Gamma priors can be integrated out in the maximum likelihood estimation. We evaluate the proposed approach, PGseq, using four real datasets and one simulated dataset, and compare its performance with other popular methods. Results show that PGseq presents competitive performance compared to other alternatives in terms of accuracy in the gene and transcript expression calculation and in the downstream differential expression analysis. Especially, we show the advantage of our method in the analysis of low expression.

## Introduction

Alternative splicing (AS) is a common phenomenon observed in eukaryotes. In this process, the multiple exons for a gene are connected in multiple ways, leading to various protein isoforms. It has been found that AS exists for more than 95% human genes [1]. Unexpected variation in AS is often associated to many diseases [2]. Therefore, the study on the variation of AS has received more and more interest in the area of biomedicine in recent years. The analysis of gene and isoform expression provides an important approach to study the variation of AS. RNA-Seq technology offers a vital tool to quantify transcript expression by generating millions of short transcript reads from an RNA population of biological samples [3]. The processing of RNA-Seq data typically involves three aspects [4]. First, reads are aligned to a reference genome or transcriptome. Second, the expressed genes and isoforms are assembled by using mapped reads. Third, given a transcriptome assembly gene and isoform expression can be calculated by counting the reads mapped to a gene and the associated isoforms. Naturally, differential expression of transcripts can also be analyzed using the obtained expression quantification. However, this paper just focuses on the third aspect in the processing of RNA-Seq data.

The expression quantification from short reads (25∼300 base pairs) is challenging. First, many reads are mapped to multiple isoforms, which belong to the same gene, since they share exons. For the paralogous genes with close sequences, it is possible to map reads to multiple genes. This read assignment uncertainty makes accurate expression quantification difficult since it is unclear which isoform a read originates if it comes from a shared exon across multiple isoforms. As read counts are proportional to the abundance of the fragments originating from a gene, the RPKM (reads per kilobase of transcript per million mapped reads) was proposed to represent the expression level of genes [5]. However, this method cannot be directly used to calculate the expression for isoforms due to read assignment uncertainty. In order to solve this problem, various Poisson-based approaches are proposed to model reads that are mapped ambiguously to multiple transcripts [6–8]. These methods utilize the additive property of the Poisson distribution to deal with the ambiguities of read mappings. Alternatively, other sophisticated approaches use probabilistic graphic models to simulate the stochastic process of generating read sequences, such as RSEM [9, 10], BitSeq [11] and the approach proposed in [12].

Second, many expression calculation approaches assume the uniform distribution of the read counts along the reference sequence, such as the RPKM representation and the Poisson-based method [6]. However, read counts follow obviously non-uniform distribution in reality due to the positional, sequencing and mappability biases [13]. This violates the uniform assumption of Poisson models. In order to obtain accurate expression estimates, these biases should be modeled and removed. Therefore, a number of bias correction strategies have been introduced to the Poisson-based models to account for the non-uniformity in read sequencing rates [8, 14, 15]. [14] used a linear model to explicitly estimate the sequencing preference of the Poisson rate at each nucleotide position based on the local surrounding sequences. [15] proposed a two-parameter generalized Poisson model for gene and exon expression computation. One parameter was used to represent expression and the other was used to model the average sequencing bias. [8] used bias curves to characterize the non-uniformity of read distributions, and incorporated these curves into a Poisson model to relieve the effects of sequencing bias. RSEM allowed the use of an empirical read start position distribution to account for the non-uniformity of the read distribution in a generative graphical model [9]. Cufflinks used a variable length Markov model to learn sequence-specific bias and positional bias based on the surrounding sequences [16]. [17] proposed a Bayesian network to predict bias at each position within a locus. The prediction can be used to adjust the biased read counts. CEM used a quasi-multinomial distribution model to capture various types of RNA-Seq biases [13]. [18] made use of the multi-sample information to jointly estimate the isoform expression and the isoform-specific read bias factor. By correcting for fragment bias, all these methods have been proved to obtain improved expression estimates showing that the bias correction is vital for accurate expression quantification. However, most of these methods explicitly estimate the average bias based on the sequence contents and position information in empirical data and do not consider the diversity of bias pattern among genes. Some approaches can model gene- or isoform-specific biases, but they use only a single point estimate to account for the average of bias properties. They therefore ignore the variability of biases across samples.

By checking the similarities among the variation patterns of read counts for individual genes, we found that the count variation is exon-specific and has the conserved pattern across different conditions for each individual gene (see the section of Materials and Methods). The same phenomenon was also observed in [14, 18]. The read sequencing preference in RNA-seq data is analogous to the probe affinities in microarray data [20–22]. In microarrays, probes have different sensitivities to the specific hybridization signals. The pattern of probe intensities varies in a gene-specific way and is also conserved for the same gene across various conditions. This characteristic has been intensively studied in the area of microarray analysis and a number of approaches have been proposed to model the probe affinity [20, 22–24]. [23] introduced the Gamma distributed latent variables to model the probe-specific sensitivity for the Affymetrix oligonuleotide arrays. This strategy has been proven to be effective in terms of both computational accuracy and efficiency.

In this paper, we propose a statistical model, PGseq (Poisson-Gamma model for RNA-Seq data), to estimate accurate gene and isoform expression by accounting for the exon-specific bias for each gene. Our approach uses Poisson distribution to model the read counts and borrows the idea in [23] of using Gamma distributed latent variables to capture the overall exon-specific read bias for each gene. An important feature of our new model is that it accounts for the distribution of the read bias instead of using a single point estimate to represent the average bias properties or explicitly depicting individual types of biases by taking into account the sequence content and position information. The bias modeled in our method is exon-specific and shared across all conditions for each individual gene, it can therefore automatically capture all the intrinsic exon-specific effects, including the sequence-specific and positional effects. Another advantage of this strategy is that the exon-specific variables representing the overall bias can be integrated out of the likelihood and this leads to an efficient maximum likelihood (ML) solution of the model. Finally, in addition to calculating gene/isoform expression our method also provides a level of uncertainty associated with these measurements. This level of uncertainty can be used to improve the downstream analysis, such as the detection of differential expression (DE).

## Materials and Methods

### Modeling distribution of exon-specific bias

**Fig 1:**
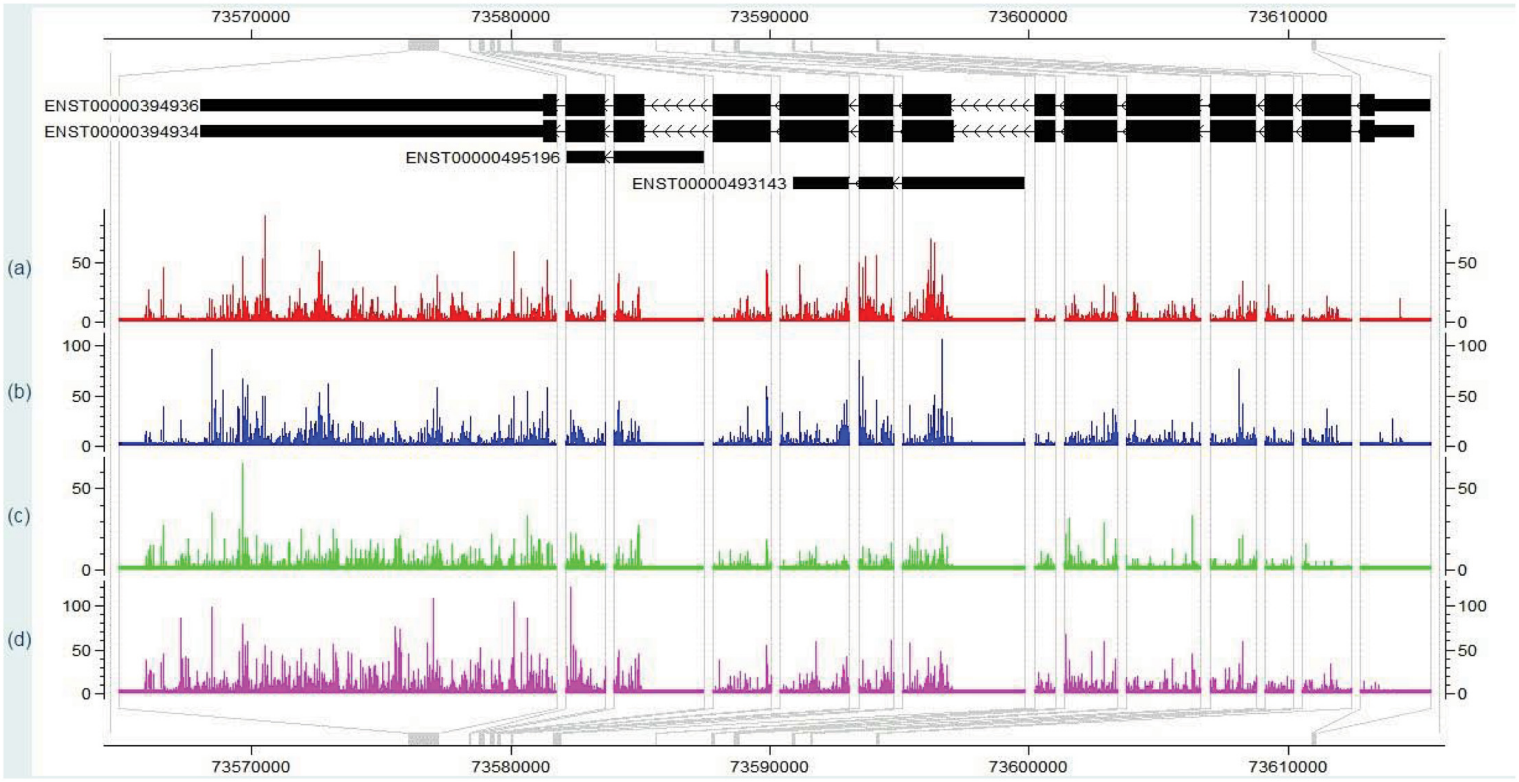
Read counts for each exonic nucleotide (nt) position in CisGenome Browser [19] along gene ENSG00000197746 (PSAP) in different tissues of the Human Body Map dataset (a) brain, (b) breast, (c) liver and (d) lung. Counts for reads starting at each exonic nucleotide position are shown.

**Fig 2:**
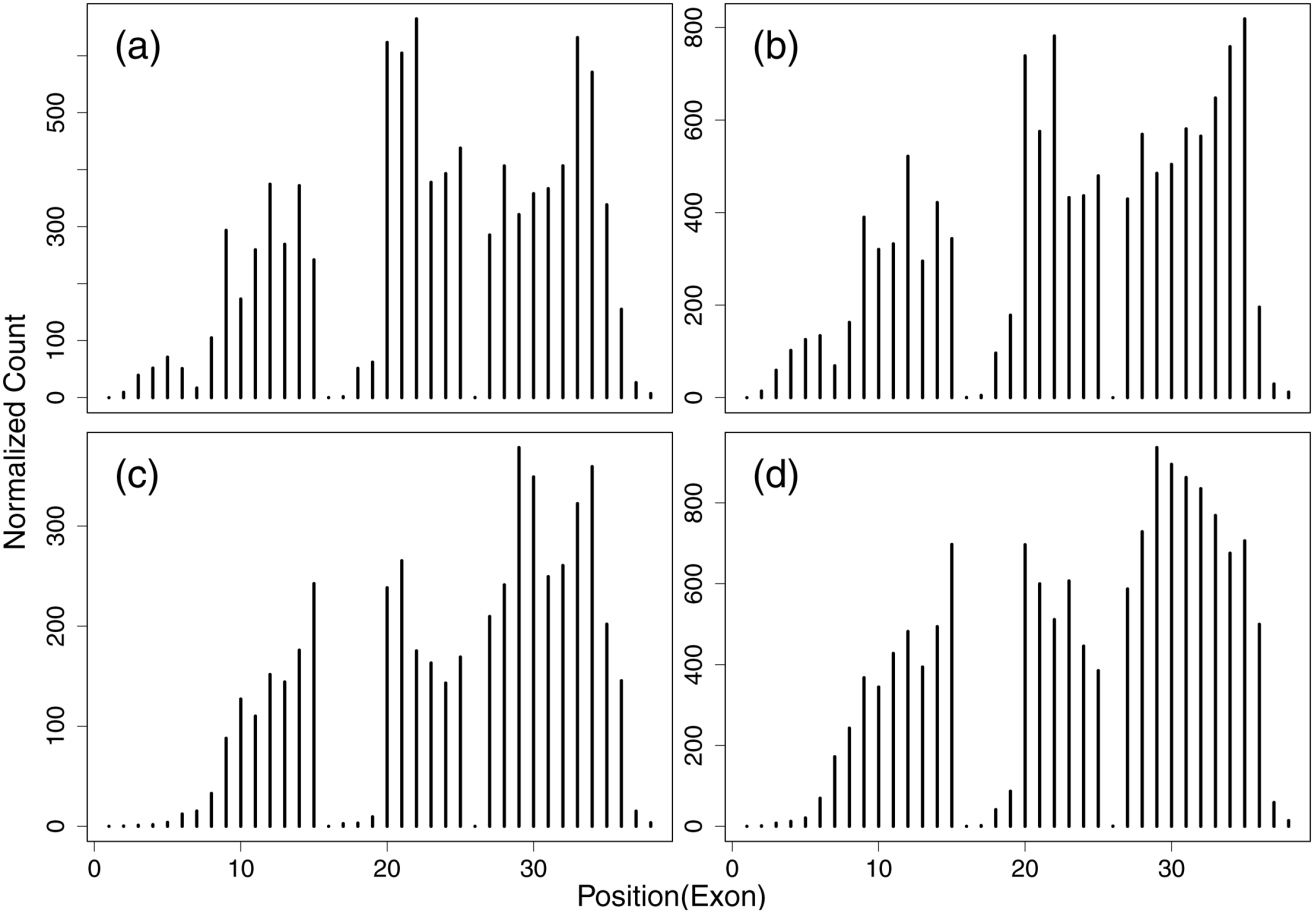
Normalized read counts for each exon along gene ENSG00000197746 (PSAP) in different tissues of the Human Body Map dataset (a) brain, (b) breast, (c) liver and (d) lung. Reads mapped to each exon are counted and normalized according to the exon length. Overlapping exons are segmented into multiple non-overlapping ones.

In RNA-Seq data analysis, it is natural to use Poisson distribution to model read counts. However, the assumption of the constant rate is violated by the serious overdispersion of read counts. Fig 1 shows the counts of a randomly selected gene ENSG00000197746 (PSAP) of four tissues from the Human Body Map project (http://www.ebi.ac.uk/arrayexpress/experiments/E-MTAB-513/). It can be seen that the counts are highly non-uniformly distributed and the variation patterns are almost consistent across the tissues. The same characteristic also holds for other genes and for other species [14]. We also plot the normalized read counts (by exon length) for each exon along this gene in different tissues as shown in Fig 2. The variation patterns are highly conserved across tissues for the normalized counts of exons. Analogously, there is high variability for the probe intensities within the same probe-set in microarray data and the variation due to probe effects is larger than the variation across technical replicates [20, 22–24]. Like the analogy with microarrrays, some exons will be preferentially sequenced due to the technical biases (e.g. GC content, secondary structure, distance from the 3’ end). There is also a more obvious reason for the low counts of some exons which is that these exons are not incorporated into transcripts as frequently. As we can see from Figs 1 and 2, the two exons with the lowest read counts in the middle part of gene ENSG00000197746 are used by a single transcript respectively, while other exons with the high read counts are included in at least two transcripts. Considering all these bias sources, we model the exon-specific sequencing preference in our approach.

We borrow the strategy of modeling the probe effects in microarray data in [23] to deal with the exon-specific fluctuation of read counts in RNA-Seq data. We believe the variability in the sequencing preference can explain the consistent overdispersion pattern across samples in the read counts. Accordingly, we introduce a latent variable *β*_*i*_ for exon *i* to describe the associated sequencing preference and share *β*_*i*_ across multiple samples. We assume that {*β*_*i*_} are independent and identically distributed random variables and are drawn from a gene-specific Gamma distribution (we ignore the subscript of gene for notation simplicity),

$$\beta_{i}∼\text{Ga}\left(a,b\right),$$ (1)

with a shape parameter *a* and a scale parameter *b*. As we can see from Fig 2 that although the overall count variation patterns are highly consistent across tissues, we can still observe the visible variability of this pattern among samples. For this reason, unlike using a single point value [13, 15, 18] to summarize all possible biases which were revealed in the previous studies [9, 14, 16, 17], we use the Gamma distribution in Eq (1) to describe the stochastic property of the gene-specific sequencing preference for each exon. It thus covers all possible types of the intrinsic sequencing biases and considers the variability of bias distribution across samples.

### The Poisson-Gamma model

For each gene, let *y*_*icr*_ represent the observed read count mapped to the *i*th exon for the *r*th technical or biological replicate under the *c*th condition. Allowing any number of isoform contributions to *y*_*icr*_, *y*_*icr*_ can be decomposed as the sum of the normalized count contributions from isoforms *t*_*icrk*_, in which exon *i* is included. We assume *y*_*icr*_ = *w*_*cr*_ *l*_*i*_∑_*k*_ *M*_*ik*_ *t*_*icrk*_, where *w*_*crl*_ and *l*_*i*_ are the scaling factors related to the sequencing depth and the exon length, respectively, and *M*_*ik*_ is defined as the indicator functions *M*_*ik*_ = 1 if exon *i* belongs to transcript *k*. Here, we assume *t*_*icrk*_ follow a Poisson distribution, *t*_*icrk*_ ∼ Pois(*β*_*i*_ *α*_*crk*_), where *β*_*i*_ is the sequencing preference of exon *i* and follows the distribution in Eq (1). We share *β*_*i*_ across all replicates and conditions in order to capture the consistent sequencing preference for each exon. The parameter *α*_*crk*_ is a quantity proportional to the abundance of transcript *k* for replicate *r* under condition *c*. Under this assumption, *y*_*icr*_ also follows a Poisson distribution,

$$y_{icr}∼\text{Pois}\;(w_{cr}l_{i}\beta_{i}\sum_{k}\;M_{ik}\alpha_{crk}).$$ (2)

The reads mapped to the exon-junction region proportionally contribute to the counts for each adjacent exon according to the fraction of the length of the mapping sequence on each exon. For paired-end data, we count the fragment between paired reads. If fragments cover multiple exons, we add the fraction of the mapping sequence length to the count of each involved exon. Also, overlapping exons are divided into multiple exons to avoid the redundant read counting. Note that MMSEQ [7] also uses a Poisson-Gamma model to fit read counts, whereas the Gamma prior is put on transcript expression and it does not consider the variability of the sequencing preference for each fragment region.

The likelihood of observed counts for a specific gene is

$$\begin{array}{cc} & L(\{y_{icr}\}|\{\alpha_{crk}\},a,b)\\ & =\prod_{i}\int \text{d}\beta_{i}P\;(\beta_{i}|a,b)\prod_{cr}P\;(y_{icr}|w_{cr}l_{i}\beta_{i}\sum_{k}M_{ik}\alpha_{crk}). \end{array}$$ (3)

The conjugacy of the Poisson-Gamma model makes the integral tractable. But there are no closed-form solution for the parameters of this model, {*α*_*crk*_}, *a* and *b*. By using the efficient optimization tool, such as donlp2 [25], the ML estimates of these parameters, $\left\{{\hat{\alpha }}_{crk}\right\}$, $\hat{a}$ and $\hat{b}$, can be easily obtained. Practically, we find that the large number of *c* × *r* can help with the estimation of the sequencing preference distribution. For the experiments where biological and technical replicates are not available, data from the multiple lanes or runs of a single library can be taken as “technical” replicates to aid the estimation of model parameters.

### Expression inference

In our model, *α*_*crk*_ represents the relative transcript abundance across conditions and replicates for the same transcript *k*. However, it cannot represent the absolute expression across transcripts belonging to different genes, as the gene-specific random variable *β*_*i*_ also accounts for the overall read count of the transcript across conditions and replicates. For this consideration, we choose to use the normalized read count *t*_*icrk*_ to represent transcript expression since it considers the effect of *β*_*i*_. This representation is different from other Poisson-based approaches for the reason that they do not adopt a gene-specific parameter to the Poisson rate as *β*_*i*_ in our model.

We assume that the normalized read counts of transcript *k* on all exons, *t*_⋅*crk*_, are independent and identically distributed random variables. With the estimated model parameters, we can obtain the distribution of *β*_*i*_, $P(\beta_{i}|\hat{a},\hat{b})$, which represents the distribution of the gene-specific sequencing preference for all exons. Considering all possible values of latent random variable *β*_*i*_, each of *t*_⋅*crk*_ follows the same distribution

$$\begin{array}{ccc} P\;(t_{icrk}) & = & \int \text{d}\beta_{i}P\;(t_{icrk}|{\hat{\alpha }}_{crk}\beta_{i})\;P\;(\beta_{i}|\hat{a},\hat{b})\\ & = & \text{NB}\;(\hat{a},\frac{{\hat{\alpha }}_{crk}}{\hat{b}+{\hat{\alpha }}_{crk}}), \end{array}$$ (4)

where NB denotes the negative binomial distribution. The expectation and variance of *t*_*icrk*_ are then

$$\langle t_{icrk}\rangle =\frac{\hat{a}}{\hat{b}}{\hat{\alpha }}_{crk}\;\text{and}\;\text{Var}\;[t_{icrk}]=\frac{\hat{a}}{{\hat{b}}^{2}}{\hat{\alpha }}_{crk}\;(\hat{b}+{\hat{\alpha }}_{crk}).$$ (5)

Assume that the normalized gene expression, *s*_*icr*_, can be calculated as the sum of the transcript contributions, i.e. *s*_*icr*_ = ∑_*k*_ *t*_*icrk*_. Similarly, the gene expression on all exons *s*_⋅*cr*_ are also assumed to be independent and identically distributed random variables, each of which follows the same distribution

$$\begin{array}{ccc} P\;(s_{icr}) & = & \int \text{d}\beta_{i}P\;(s_{icrk}|\sum_{k}\;{\hat{\alpha }}_{crk}\beta_{i})\;P\;(\beta_{i}|\hat{a},\hat{b})\\ & = & \text{NB}\;(\hat{a},\frac{\sum_{k}{\hat{\alpha }}_{crk}}{\hat{b}+\sum_{k}{\hat{\alpha }}_{crk}}). \end{array}$$ (6)

The expectation and variance of *s*_*icr*_ are therefore

$$\langle s_{icr}\rangle =\frac{\hat{a}}{\hat{b}}\sum_{k}\;{\hat{\alpha }}_{crk}\;\text{and}\;\text{Var}\;[s_{icr}]=\frac{\hat{a}}{{\hat{b}}^{2}}\sum_{k}\;{\hat{\alpha }}_{crk}\;(\hat{b}+\sum_{k}\;{\hat{\alpha }}_{crk}).$$ (7)

Using sampling from the negative binomial distributions in Eqs (4) and (6), the expectation and variance of the logarithmic transcript/gene expression can be obtained. This expression representation is useful for propagating the measurement error in the subsequent downstream analyses of both gene and transcript expression.

Note that the transcript and gene expression are both expressed as negative binomial distributions here. The two-parameter NB distribution has been thought to be advantageous for modeling the read counts in the differential expression analysis of RNA-Seq data due to its ability to model the overdispersion in read distributions [26–28]. The expression in Eq (7) has the similar parametrization as the NB model, sSeq, proposed in [28]. The expected expression *μ* and the dispersion parameter *ϕ* in sSeq are analogous to $\frac{a}{b}\alpha$ and $\frac{1}{a}$, respectively, in our method. However, our approach is different from sSeq and other NB-based approaches. First, instead of modeling the distribution of the whole count for each gene we model the variability of the count for each individual exon. Consequently, it is possible to estimate the gene-specific bias distribution and to apply the ML method for parameter estimation. Second, we decompose the total count for each exon into a sum of the contributions from related transcripts and thus obtain the expression for each transcript which can be useful for downstream transcript level analyses.

### Software

The proposed PGseq method is implemented in Python and C. After aligning the primary reads to the reference transcriptome by Bowtie 2 [29], the alignment files are then processed using our Python scripts to obtain the read counts for each exon. We employ the fast optimization toolkit, donlp2 [25], to estimate the model parameters. Both parameter optimization and expression inference are implemented in fast C codes. We also make use of parallel computing to improve the computation efficiency. The software and documentation are freely available online from the website https://github.com/PUGEA/PGSeq.

### Datasets

We evaluate the proposed approach, PGseq, on the estimation of gene and isoform expression using three real datasets and one simulated dataset, and considering both single-end and paired-end data.

We use the well studied Microarray Quality Control (MAQC) dataset [30] to validate the expression estimation from PGseq at gene level. MAQC project measured gene expression from high-quality RNA samples to assess the comparability across multiple platforms. This dataset has been widely used as the benchmark to verify various analysis methods [31–33]. We select two RNA samples, the universal human reference (UHR) RNA and the human brain reference (HBR) RNA, from the Illumina platform. The Short Read Archive accession number is SRA010153 for single-end data and SRA012427 for paired-end data. Around one thousand genes have been measured by the qRT-PCR experiments and can be served as the gold standard to benchmark the gene expression estimation obtained from other platforms. We used the Ensembl annotation data (NCBI37/hg19) and obtained 841 matching qRT-PCR validated genes. Among these qRT-PCR validated genes, we use the method in [32] to filter 217 DE genes and 88 non-DE genes with high confidence according to the qRT-PCR measurements. Data of these 305 qRT-PCR validated genes is used as a gold standard to evaluate the sensitivity and the specificity of various DE analysis approaches.

A real human colorectal cancer (HCC) dataset [34] is also used to further validate the gene expression estimation of PGseq. In this dataset, the fluorouracil-resistant (MIP101) and -nonresistant (MIP/5-FU) human colorectal cancer cell lines were investigated using paired-end RNA-seq experiments. Since there are no biological or technical replicates in this dataset, we select seven lanes for each condition and take them as seven “technical” replicates in order to obtain better estimation of model parameters. For each replicate we use about 9 million reads. Reads are aligned using Ensembl annotation data (NCBI36/hg18). There are 192 genes which were quantified by the qPCR experiments in this dataset. The number of qPCR validated genes is reduced to 101 by merging redundancy and being successfully mapped to the reference. The qPCR measurements of these 101 genes are used to validate the gene expression estimates of our method. Among these genes, we use the similar selection method in [28] to choose 21 DE genes and 14 non-DE genes with high confidence. Data of these 35 genes is also used as a gold standard to compare methods in DE analysis.

A publicly available human breast cancer (HBC) dataset [35] is used to validate the estimation of transcript expression of PGseq. This dataset contains single-end data and includes two conditions, the human breast cancer cell line (MCF-7) and the normal cell line (HME). Four genes (TRAP1, ZNF581, WISP2 and HIST1H2BD) which contain multiple transcripts were validated using the qRT-PCR experiments [36]. Two transcripts for each gene have been interrogated for both cell lines. We used the UCSC knownGene transcriptome annotation (NCBI36/hg18) for obtaining all annotation information for the eight qRT-PCR validated transcripts.

Since the true expression for a large number of transcripts are not available, we generated simulated data using our model based on the calculated parameters from the qRT-PCR validated genes of HBR sample in MAQC dataset. This dataset is mainly used for sanity checking of our method. Around 100 million reads are generated individually for each of the seven “technical” replicates. Since we simulate data of a single condition, we omit the subscript *c* in the following mathematical symbols. For each gene, we first sample *β*_*i*_ from $\text{Ga}(\hat{a},\hat{b})$ and *α*_*rk*_ from $\text{N}({\hat{\alpha }}_{1k},{\hat{\alpha }}_{1k}/20)$, where ${\hat{\alpha }}_{1k}$ is the estimated parameters for the first replicate. The count for each exon, *y*_*ir*_, is then drawn from $\text{Pois}(w_{l}l_{i}{\hat{\beta }}_{i}\sum_{k}M_{ik}{\hat{\alpha }}_{rk})$. Reads with count *y*_*ir*_ are then sequenced from the reference sequence by considering the start position along the reference. If *y*_*ir*_/*l*_*i*_ > 0.1, reads are sequenced according to the true histogram in the real dataset. Otherwise, reads are uniformly sequenced. The length of the sampled fragments is 35 base pairs for the sing-end dataset, and sampled from N(206, 19.6) for the paired-end dataset. The sequenced read length for both ends of paired-end data is 50. The constants used in the above distributions are chosen based on empirical data. The sampled reads hold the consistent realistic non-uniformity in the distribution across technical replicates.

Finally, we use the human brain dataset (HBD) downloaded from DDBJ [37] with accession number SRA009447 to show the use of our method for datasets with biological replicates. We also use this dataset to verify the performance of our approach for lowly expressed genes. HBD dataset includes two conditions, the adult and fetal human brains, each of which contains three biological replicates. For each biological replicate, two or three technical replicates were used. We pool reads from technical replicates for each biological replicate and make expression estimation from the six pooled sets.

## Results

We compare PGseq with other three popular alternatives, Cufflinks v2.2.1, RSEM v1.2.19 and MMSEQ v1.0.7, for gene and transcript expression estimation. All these softwares are used with default parameters. We use the MAQC, HCC and simulation datasets to evaluate the performance of PGseq on gene expression estimation. The HBC and simulation datasets are used to verify the accuracy of our method for isoform expression estimation. Finally, we apply PGseq to DE analysis and compare it with other competitive approaches.

### Transcript expression deconvolution

**Fig 3:**
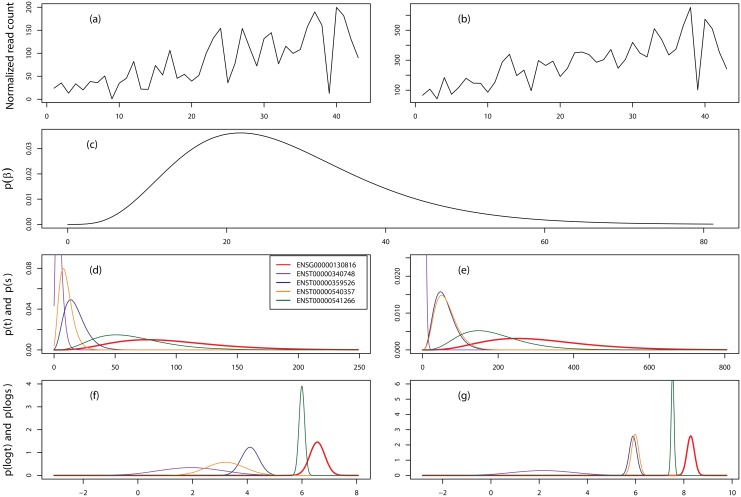
Transcript expression deconvolution by PGseq for gene ENSG00000130816. (a) and (b) show the normalized read counts for samples HBR and UHR, respectively, for each exon. (c) shows the estimated gene-specific read distribution. The 3rd panel shows the obtained NB distributions for both gene and isoforms for the two samples. The last panel presents the approximated Gaussian distributions of logged gene and isoform expression. This gene contains four isoforms and the total normalized read counts for both samples are 601 and 2341, respectively.

**Fig 4:**
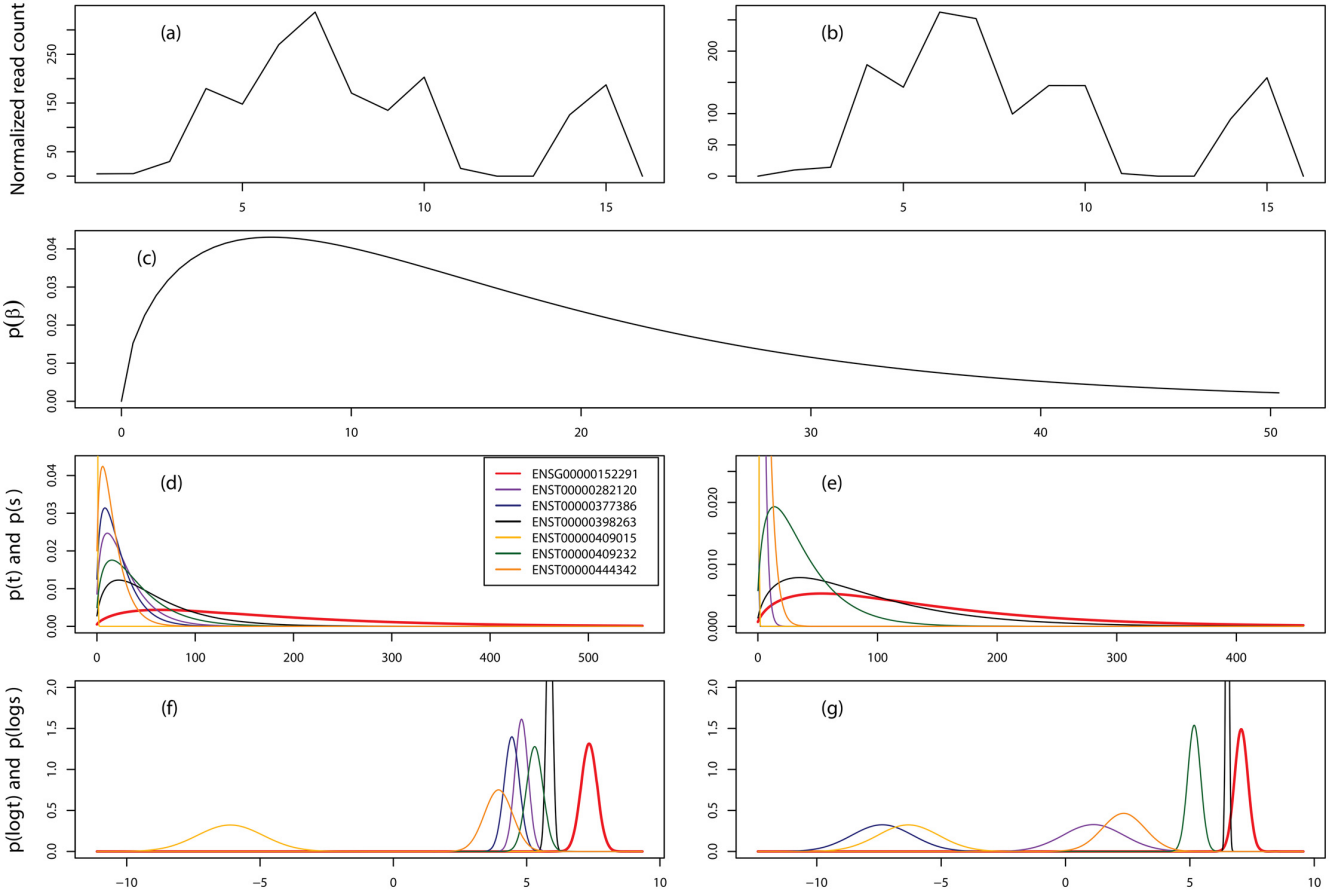
Transcript expression deconvolution by PGseq for gene ENSG00000152291. (a) and (b) show the normalized read counts for samples HBR and UHR, respectively, for each exon. (c) shows the estimated gene-specific bias distribution. The 3rd panel shows the obtained NB distributions for both gene and isoforms for the two samples. The last panel presents the approximated Gaussian distributions of logged gene and isoform expression. This gene contains six isoforms and the total normalized read counts for the two samples are both 1321.

The major advantage of PGseq is that it is able to deconvolute the read counts for a gene to obtain the individual NB distributed isoform expression by considering the gene-specific read bias distribution. Before evaluating the accuracy of expression measurements estimated from our method, we randomly select two examples, genes ENSG00000130816 and ENSG00000152291, from the MAQC SRA010153 data to show this advantage as presented in Figs 3 and 4, respectively. As we can see that these two genes present very different count variation patterns. Consequently, we obtain the different bias distributions as shown in subplots (c) in both figures. We believe the obtained bias distributions are able to capture the gene-specific count variation patterns for individual genes. We note that gene ENSG00000130816 is up-regulated in sample UHR while gene ENSG00000152291 is invariant across the two samples by comparing the observed total read counts for both samples. From Fig 3 we can find that even though the gene is obviously differentially expressed, isoform ENST00000340748 is low expressed and largely invariant across the two samples while the other three are up-regulated in sample UHR. Fig 4 indicates that the gene expression is unchanged while many isoforms are differentially expressed across the two samples. The examples here show that a reasonable approach, which is able to accurately deconvolute transcript expression, is vital for the investigation of AS variation.

### Validation of gene expression estimation

The SRA010153 data in the MAQC dataset contains two RNA samples, HBR and UHR. Each sample includes seven lanes which can be seen as seven “technical” replicates. The SRA012427 data contains three technical replicates for the single sample UHR. We apply all approaches to each replicate and compute the average gene expression for each sample. All the methods are run with the bias correction mode turned on. The squared Pearson correlation coefficient (*R* ^2^) of the logarithmic average gene expression estimates with the logarithmic qRT-PCR measurements for the 841 mapping qRT-PCR validated genes are calculated as shown in the first three rows in Table 1. We can see that PGseq outperforms the other three alternatives for the comparisons on all three samples. Notice that Cufflinks, RSEM and MMSEQ all obtain less consistent results with the qRT-PCR results for sample UHR using the paired-end data than the single-end data. This contradicts the common sense that the paired-end protocol is more advantageous than the single-end protocol and thus should lead to more accurate expression measurements. We find that only PGseq produces more accurate expression estimates for the paired-end data than the single-end data. The fourth and fifth rows show the calculation accuracy of various methods at gene level against the qPCR measurements for the two samples in the HCC dataset. For this dataset, PGseq performs as almost equally well as other approaches for sample MIP101, but significantly better for sample MIP/5-FU. Similarly, the last two rows show the comparison results of expression estimation accuracy against the ground truth at gene level using the simulated datasets. Since the data is generated from our model, it is not surprising that PGseq obtains the most accurate results than other approaches. This shows the consistency of our model. Note that although the data is biased to PGseq, the other three methods also present the high accuracy on gene expression calculation showing good performance of these approaches on gene expression calculation.

**Table 1:** Comparison of expression estimation accuracy at gene level using various datasets.

**Dataset**	**Cufflinks**	**RSEM**	**MMSEQ**	**PGseq**
MAQC.HBR.SE	0.812	0.808	0.800	**0.845**
MAQC.UHR.SE	0.837	0.840	0.832	**0.854**
MAQC.UHR.PE	0.723	0.800	0.805	**0.860**
HCC.MIP101.PE	0.770	**0.785**	0.779	0.776
HCC.MIP/5-FU.PE	0.844	0.853	0.852	**0.881**
Simulation.SE	0.930	0.974	0.974	**0.979**
Simulation.PE	0.950	0.981	0.980	**0.984**

For the MAQC and HCC datasets, the *R* ^2^ correlation coefficients of the logarithmic average expression for the matching PCR-validated genes with the logarithmic qRT-PCR or qPCR results are calculated. Two samples (HBR and UHR) in single-end (SE) data (SRA010153) and one sample (UHR) in paired-end (PE) data (SRA012427) are used for the MAQC dataset. Seven lanes with 9 million paired-end reads for each lane are used for the HCC dataset. For the simulated dataset, the *R* ^2^ correlation coefficients of the estimated gene expression with the ground truth are calculated. Both single-end and paired-end simulated data are used. The best result for each comparison is highlighted in bold.

We have shown that PGseq provides better correlation with the qRT-PCR findings. Next, we divide the 841 qRT-PCR validated genes in the MAQC data set into three groups with low, medium and high expression. The genes with qRT-PCR measurement below 0.02 are assigned to the “low” group, 0.02 and 0.2 to the “medium” group and above 0.2 to the “high” group. For each group we examine the correlation between the calculated gene expression with the qRT-PCR measurements to reveal the performance of our approach for individual group with low, medium and high expression, respectively. Table 2 shows the correlation of each method for each group. We can see that PGseq presents the outstanding performance for the low group, while obtains moderate results for the medium and high groups. This testifies that the overall outperformance of PGseq is mainly due to its superiority in low expression. Normally, it is difficult to measure expression of genes with low read counts because there is usually high level of noise associated to these genes [38].

**Table 2:** Comparison of gene expression estimation accuracy for groups with different level of expression.

**Dataset**		**Cufflinks**	**RSEM**	**MMSEQ**	**PGseq**
Low	MAQC.HBR.SE(282)	0.333	0.326	0.303	**0.492**
	MAQC.UHR.SE(258)	0.257	0.268	0.250	**0.382**
	MAQC.UHR.PE(282)	0.203	0.328	0.357	**0.530**
Medium	MAQC.HBR.SE(246)	0.452	0.499	0.472	**0.520**
	MAQC.UHR.SE(246)	**0.480**	0.473	0.446	0.406
	MAQC.UHR.PE(246)	0.399	0.480	0.489	**0.533**
High	MAQC.HBR.SE(313)	**0.707**	0.694	0.673	0.663
	MAQC.UHR.SE(337)	0.690	**0.719**	0.693	0.693
	MAQC.UHR.PE(313)	0.658	**0.682**	0.678	0.649

For the MAQC dataset, the *R* ^2^ correlation coefficients of the logarithmic average expression measurements with the logarithmic qRT-PCR results. Data are divided into the “low”, “medium” and “high” groups according to the level of the qRT-PCR measurements. The number after each dataset shows the number of genes belonging to this group. The best result for each comparison is highlighted in bold.

### Validation of transcript expression estimation

We use a real human breast cancer dataset with qRT-PCR validation to verify the transcript expression measured from PGseq. The qRT-PCR measurements for eight transcripts under the two conditions are taken as the gold standard to compare the performance of various methods. We calculate *R* ^2^ correlation coefficients between the obtained logarithmic transcript expression with the logarithmic qRT-PCR measurements. The consistency of results between various approaches with qRT-PCR experiments is shown in the first row in Table 3. We can see that PGseq obtains the most consistent results with the qRT-PCR measurements as compared with other alternatives.

**Table 3:** Comparison of expression estimation accuracy at transcript level.

**Dataset**	**Cufflinks**	**RSEM**	**MMSEQ**	**PGseq**
HBC	0.558	0.615	0.578	**0.657**
Simulation.SE	0.724	0.768	0.738	**0.853**
Simulation.PE	0.831	0.862	0.836	**0.900**

For the HBC dataset, the *R* ^2^ correlation coefficients of the logarithmic average expression measurements with the logarithmic qRT-PCR results for the eight validated transcripts under the two conditions are calculated. For the simulated dataset, the *R* ^2^ correlation coefficients with the ground truth are calculated. Both single-end and paired-end simulated data are simulated. The best result for each comparison is highlighted in bold.

We then use the simulated datasets to verify the transcript expression estimation of our method for a larger number of transcripts. The last two rows in Table 3 show the comparison results of expression estimation accuracy against the ground truth at transcript level. It can be found that all the four methods obtain more consistent results with the ground truth using the paired-end data than the single-end data. However, the obtained *R* ^2^ values are much lower than those for gene expression calculation in Table 1. This shows that the computation of transcript expression is much more difficult than that of gene expression. We find that PGseq outputs the highest accuracy for both comparisons and the superiority for the single-end data is especially significant. Note that both PGseq and MMSEQ are Poisson-Gamma models, and the difference between these two models is that PGseq considers the variability of the gene-specific read sequencing preference for each exon while MMSEQ does not. The comparison results demonstrate that properly modeling the distribution of the sequencing preference contributes to the estimation of transcript expression.

### Propagating measurement error in DE analysis

Our method is also advantageous for providing measurement error in expression estimates. Propagating measurement uncertainty in the downstream analyses has been approved to obtain biologically more relevant results for microarray data analyses [39–41]. The recent study in [42] has also shown that accounting for posterior uncertainty in expression measurements can improve the power of DE analysis of RNA-seq data. We make use of the proposed DE analysis method, MMDiff, in [42] to show the usefulness of the measurement error obtained by PGseq in DE analysis.

**Fig 5:**
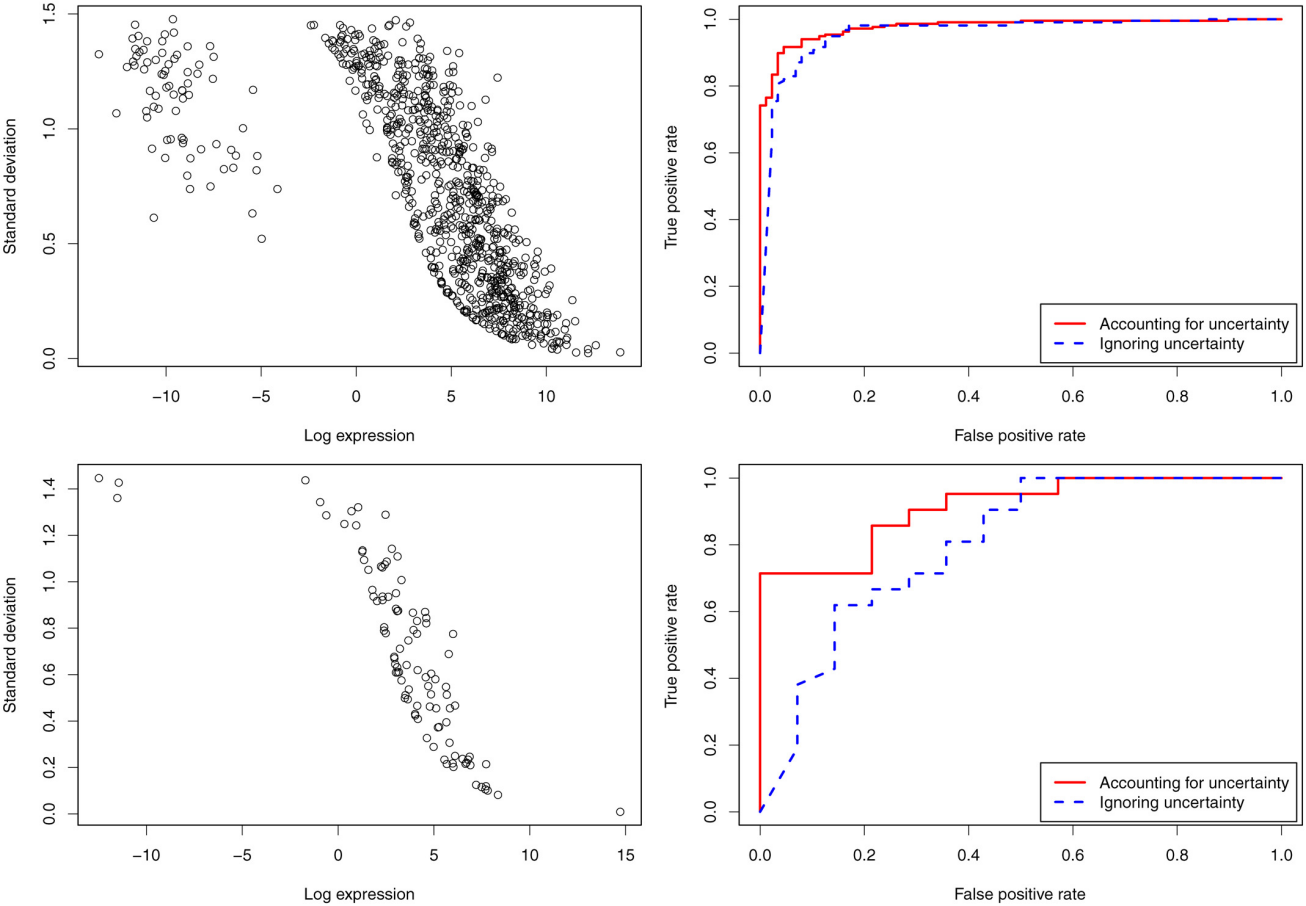
Usefulness of measurement error obtained by PGseq. The scatter plots (left column) show the standard deviation vs. the logarithm of gene expression for the PCR-validated genes in the MAQC (upper panel) and HCC (lower panel) datasets. As the expression level increases the associated measurement error decreases. The ROC curves (right column) indicate the difference between accounting for and ignoring measurement uncertainty in the DE analysis. The DE analysis employs MMDiff which considers expression measurement error. The solid curves show the performance of the DE analysis considering expression measurement error, and the dashed lines ignore measurement error by setting zero measurement error.

The left column in Fig 5 shows the scatter plots of the standard deviation vs. the logarithmic gene expression calculated from PGseq for the PCR-validated genes in the MAQC and HCC datasets. It can be seen that as the expression increases the measurement error decreases. For the genes with logged expression below -5, the measurement error does not increase accordingly. By investigating the raw read data, we find that the read counts related to these genes for all the samples are close to zero, the obtained low expression estimates are then associated with relatively high certainty. In order to show the usefulness of the calculated measurement error of PGseq, we make use of MMDiff, which is able to propagate expression measurement error to the DE analysis, and combine PGseq and MMDiff to produce receiver operator characteristic (ROC) curves for the true DE genes and the non-DE genes by considering the measurement error and ignoring this error. The comparison results are shown in the right column in Fig 5. By considering the measurement error calculated from PGseq, we obtain the better ROC curves for both datasets than ignoring measurement error (i.e. setting zero measurement error). The area under the ROC curve (AUC) for the MAQC dataset is 0.959 if ignoring the measurement error, while 0.977 if considering the measurement error. For the HCC dataset, AUC values for ignoring and considering the measurement error are 0.801 and 0.912, respectively. This demonstrates that the obtained measurement error from PGseq significantly helps with the downstream DE analysis.

**Fig 6:**
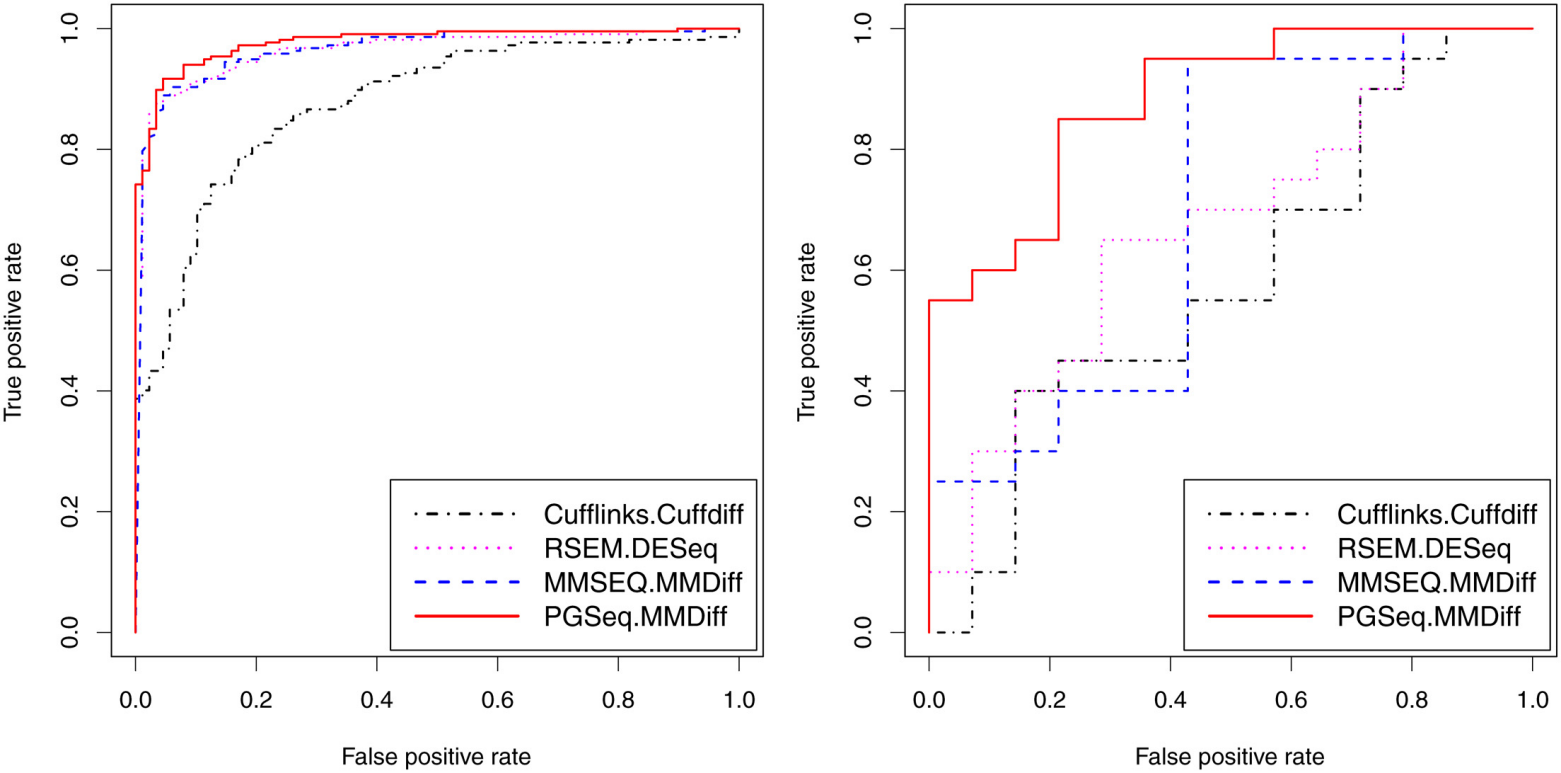
ROC curves of DE analysis for the selected PCR-validated genes in the MAQC (left) and HCC (right) datasets. Cufflinks and MMSEQ are combined with the corresponding embedded DE analysis methods, Cuffdiff and MMDiff, respectively. RSEM is combined with DESeq. PGseq is combined with MMDiff for propagating measurement error in the DE analysis.

We produce the ROC curves for various combination of the expression estimation methods and the DE analysis approaches as shown in Fig 6. The corresponding AUCs for the MAQC and HCC datasets are shown in Table 4. Cufflinks and MMSEQ are combined with their own embedded DE methods, Cuffdiff [43] and MMDiff, respectively. The embedded DE method of RSEM is EBSeq [44]. We find that DESeq [26] combined with RSEM obtains better results than EBSeq (data not shown) and we thus choose DESeq as the DE method for RSEM in the following comparisons. The combination of PGseq and MMDiff obtains higher accuracy than other combinations for both datasets. Note that even though using the same DE analysis method, PGseq still outperforms MMSEQ. Since PGseq and MMSEQ are both Poisson-Gamma models, we believe that the difference in the performance is due to the fact that PGseq models the distribution of exon-specific sequencing bias while MMSEQ does not take this into consideration. Comparisons in this section show that modeling bias distribution in expression estimation can lead to improved DE analysis results for RNA-seq data.

**Table 4:** Area under ROC curves for detection of DE genes.

**Dataset**	**Cufflinks (Cuffdiff)**	**RSEM (DESeq)**	**MMSEQ (MMDiff)**	**PGseq (MMDiff)**
MAQC	0.876	0.965	0.965	**0.977**
HCC	0.422	0.725	0.757	**0.912**

Cufflinks, RSEM and MMSEQ are combined with the corresponding embedded DE analysis methods, Cuffdiff, DESeq and MMDiff, respectively. PGseq is combined with MMDiff for propagating measurement error in the DE analysis.

### Finding DE for lowly expressed genes

**Fig 7:**
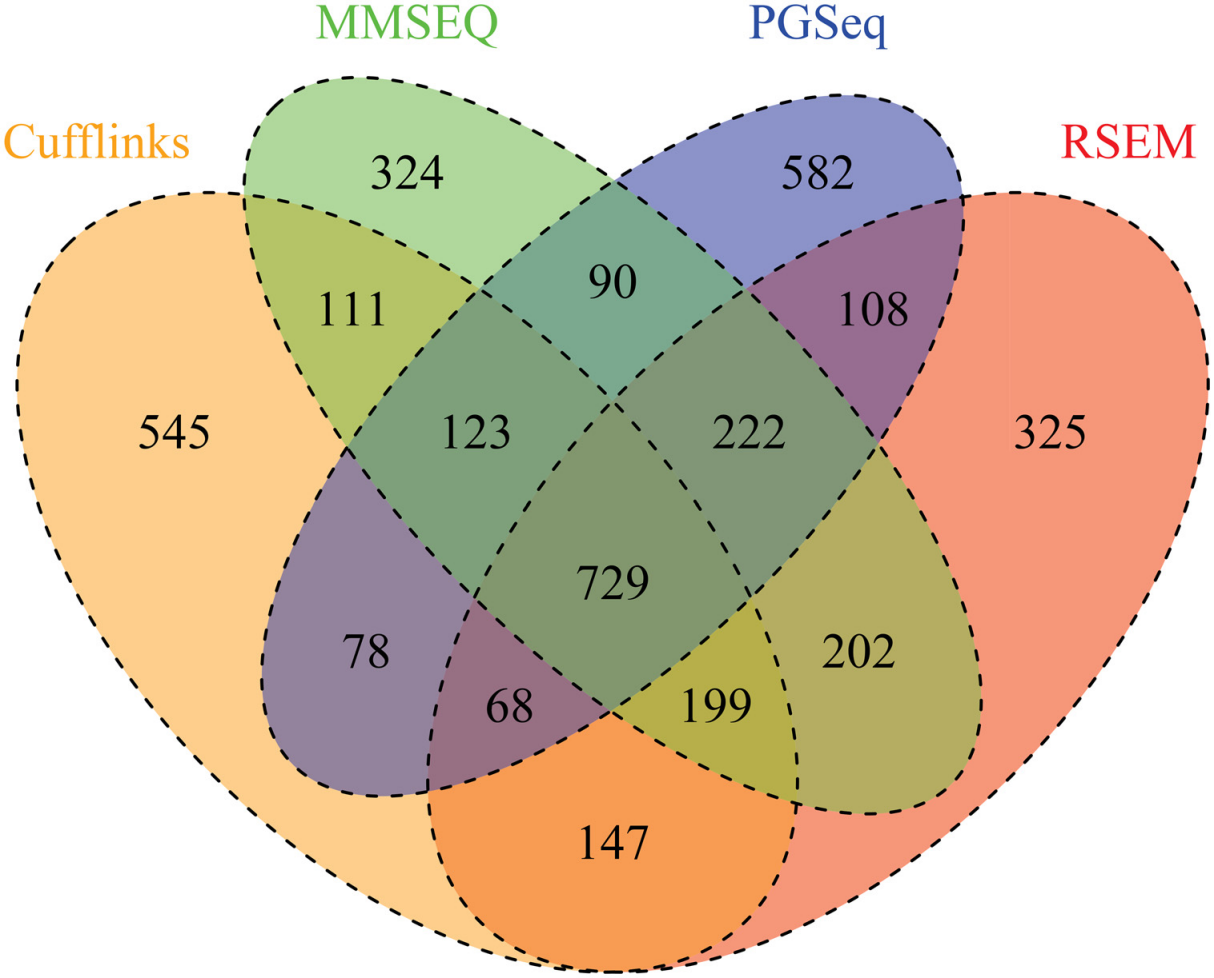
Venn diagram of the significant DE genes for the HDB dataset. The big ovals represent the number of the significant DE genes found by the four methods: Cufflinks, MMSEQ, PGseq and RSEM, which combined with CuffDiff, MMDiff, MMDiff and DESeq, respectively. The overlap of the four ovals in the middle of the diagram is 729, which is the number of the DE genes found by all of the four approaches.

**Fig 8:**
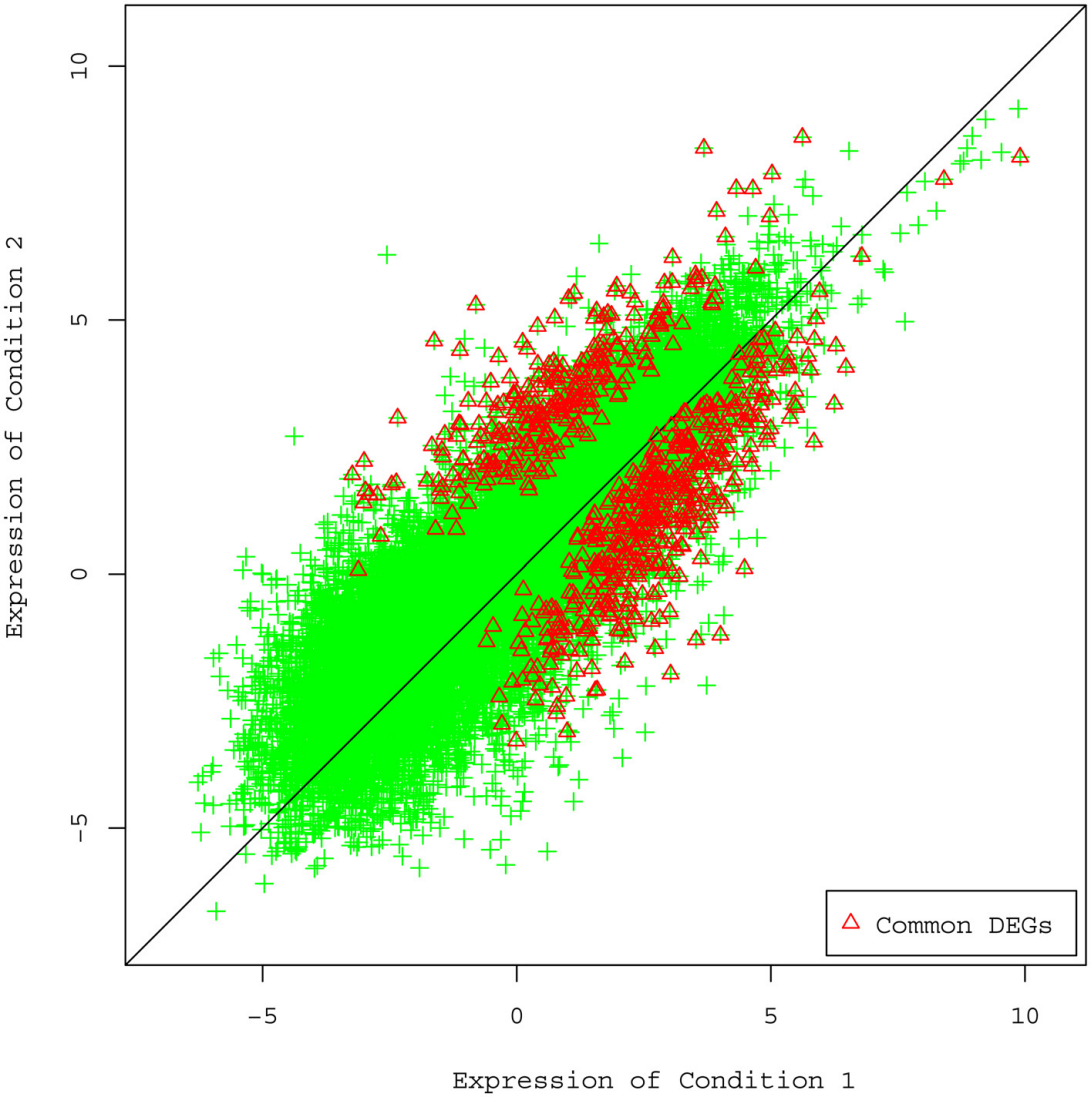
Scatter plot of the average logged RPKM estimation for the HDB dataset. There are 23,402 expressed genes (RPKM>0 for both conditions), among which the 729 common DE genes found by all the four DE methods are represented by red triangles and others by green crosses.

Finally, we consider a real HBD dataset which includes biological replicates. In this dataset, we pool the technical replicates and consider only biological replicates. We apply the above four combined methods to this dataset for DE detection. Since these methods use the different statistics for significance test, the significant levels are not comparable. Hence, for each method we select the top 2,000 genes in the significance ranking of differential expression. Fig 7 shows the Venn diagram of the significant DE genes found by the four approaches. It can be seen that there are quite a number of common DE genes found by any pair of these methods. We found 729 genes which are declared DE by all the four methods. We then plot the scatter plot of the average logged RPKM estimation as shown in Fig 8. We find that the majority of the 729 common DE genes distribute over the medium and high expression areas and few are found in the lower end. It shows the obvious difficulty of detecting DE for lowly expressed genes.

**Fig 9:**
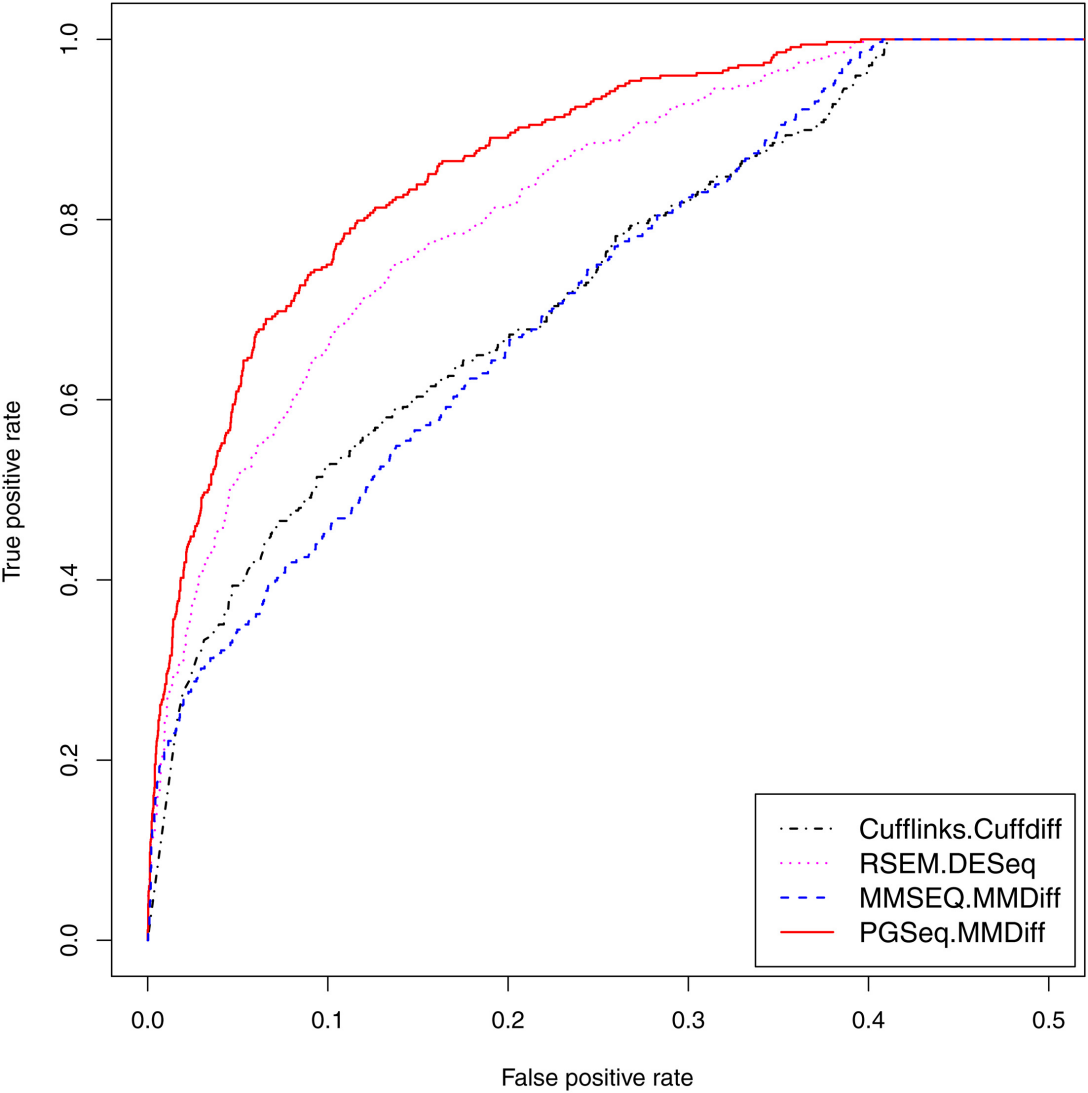
ROC curves of DE analysis for the lowly expressed genes in the HBD datasets. The 348 DE genes found by all the four methods are taken as the “true” DE genes and the rest as non-DE genes.

We have demonstrated in previous section that PGseq obtains more accurate expression estimation for lowly expressed genes. Here, we use the HBD dataset to show the power of our approach in the DE detection for the low expression. We filter the lowly expressed genes with expression between 0.01 and 2.0 (measured in RPKM) to obtain 10,157 low expression genes. We apply the four combined methods to these genes for the DE detection. For each method we find a set of 2,000 most significant DE genes. The union of these four sets contains 4,373 genes, each of which appear at least once in the four sets. Correspondingly, we find 348 genes in the intersection which is the overlap of these four sets. We take these 348 genes as the significant DE genes since all of the four approaches find them as significant DE genes, and the rest among the 4,373 genes as the non-DE genes. We use this data to draw ROC curve for each method to show its power in finding “true” lowly expressed DE genes. The higher the curve, the more powerful the related method in finding DE genes in low expression area. Fig 9 shows the ROC curves for the four DE approaches. We can see that PGseq combined with MMDiff presents the highest power in the DE detection. This example shows again that our approach has the advantage in the analysis of low expression genes over other alternatives.

In addition, we leave one method out and find the “true DE set”, validated by three methods each time, among the 4,373 genes. For the four sets of the “true DE genes”, we perform the comparisons for all the four approaches respectively and show the results in S1 Fig. It can be seen that our approach shows competitive strength in plots (a) ∼ (c). For plot (d) where the DE genes are agreed by the three alternative methods except PGseq, our method fails to find many “true positives”. When we examine the distribution of the “true DE genes” for each plot as shown in S2 Fig, we find that although plot (d) contains more “DE genes” than other plots but there are many genes locating close to the diagonal and these genes are likely false positives. In contrast, there are few false positives on the other three plots.

## Discussion

In this manuscript, we proposed a Poisson model to fit the read counts for each gene and use the gene-specific Gamma-distributed latent variables to capture the variability of the read sequencing preference for every exon. The bias property modeled in our method is shared across all conditions for each individual gene, and automatically captures all the intrinsic exon-specific effects. We used four real datasets and one simualted dataset to verify the performance of our method and compared it with other popular alternatives, Cufflinks, RSEM and MMSEQ. For the real datasets, we calculated the *R* ^2^ correlation coefficients of the estimated gene and transcript expression with the PCR measurements, and performed DE analysis to show the advantages of our approaches. For the simulated dataset, the consistence with the ground truth was also compared. The comparison results have shown that the proposed PGseq approach obtains competitive results for most comparison cases and performs especially better for lowly expressed genes.

Our work indicates that the non-uniformity of read distribution is one of the most important characteristics of RNA-seq data and appropriately modeling the sequencing bias can remarkably improve the accuracy of the expression calculation. We merged all possible biases in the read sequencing into an exon-specific random variable and did not look into any specific sequence content around any specific position as many methods did. A Gamma prior was put on this variable and it was integrated out in the ML estimation. Therefore, all possible values of this variable were considered in the bias correction. This is distinct from many other methods which explicitly calculate the point estimate for each of gene- or isoform-specific biases. Our approach seems desirable based on the comparison results in this manuscript.

Another advantage of our method is that PGseq is able to provide a level of uncertainty associated with the gene and transcript expression estimates. This level of uncertainty can be propagated to the downstream analysis and obtain improved analysis results. We combined our method with a recently proposed DE analysis method, MMDiff, which incorporates measurement error of expression estimates to improve DE analysis. We evaluated this approach using two real PCR-validated datasets for DE analysis. The obtained ROC curves showed that our method significantly outperforms other popular combinations for finding differentially expressed genes. This demonstrates the usefulness of the measurement error provided by our method in downstream analysis.

We assumed that the exon-specific read variation pattern is conserved across multiple samples. We therefore shared the bias distribution across all samples to capture this pattern. In the application to the datasets in this manuscript, our method processed all samples in a single run. In case significant biological variance violates this assumption, the model can be applied sample by sample to estimate the sample-specific bias distribution. For each run, it would be helpful to model estimation if considering as much replicate information as possible. Practically, biological replicates are preferred to be considered. If biological replicates are not available or the individual sample-specific bias is of interest, the multiple lane information for a single library can also be considered. Finally, we mainly applied our method to data from human genome which have a relatively large number of splicing, we thus modeled the bias variation for each exonic position in order to have an appropriate population size for estimating bias distribution. For simpler genome, such as yeast, where many genes do not have lots of exons, the positions of interest along the reference sequence are not necessary exonic. They can be any sub-sequences of short length. In that case, a proper segmentation of the reference sequence would be needed.

## Supporting Information

S1 Fig ROC curves from the four approaches for datasets validated by three methods. The datasets are validated by the three methods except (a) Cufflinks, (b) RSEM, (c) MMSEQ and (d) PGSeq.
(PDF)

S2 Fig Scatter plots of the averaged logged RPKM estimation for lowly expressed genes in HDB dataset. The common DE genes (represented by red triangles) are agreed by three methods except (a) Cufflinks, (b) RSEM, (c) MMSEQ and (d) PGSeq, respectively. Others are represented by green crosses.
(PDF)
