# Supplementary material for: Modeling Exon-Specific Bias Distribution Improves the Analysis of RNA-Seq Data
Source: PLoS One. 2015 Oct 8;10(10):e0140032. doi: 10.1371/journal.pone.0140032 (PMC4598124; doi:10.1371/journal.pone.0140032)
Supplement: S1 Fig — The datasets are validated by the three methods except (a) Cufflinks, (b) RSEM, (c) MMSEQ and (d) PGSeq. (PDF) [file pone.0140032.s001.pdf]

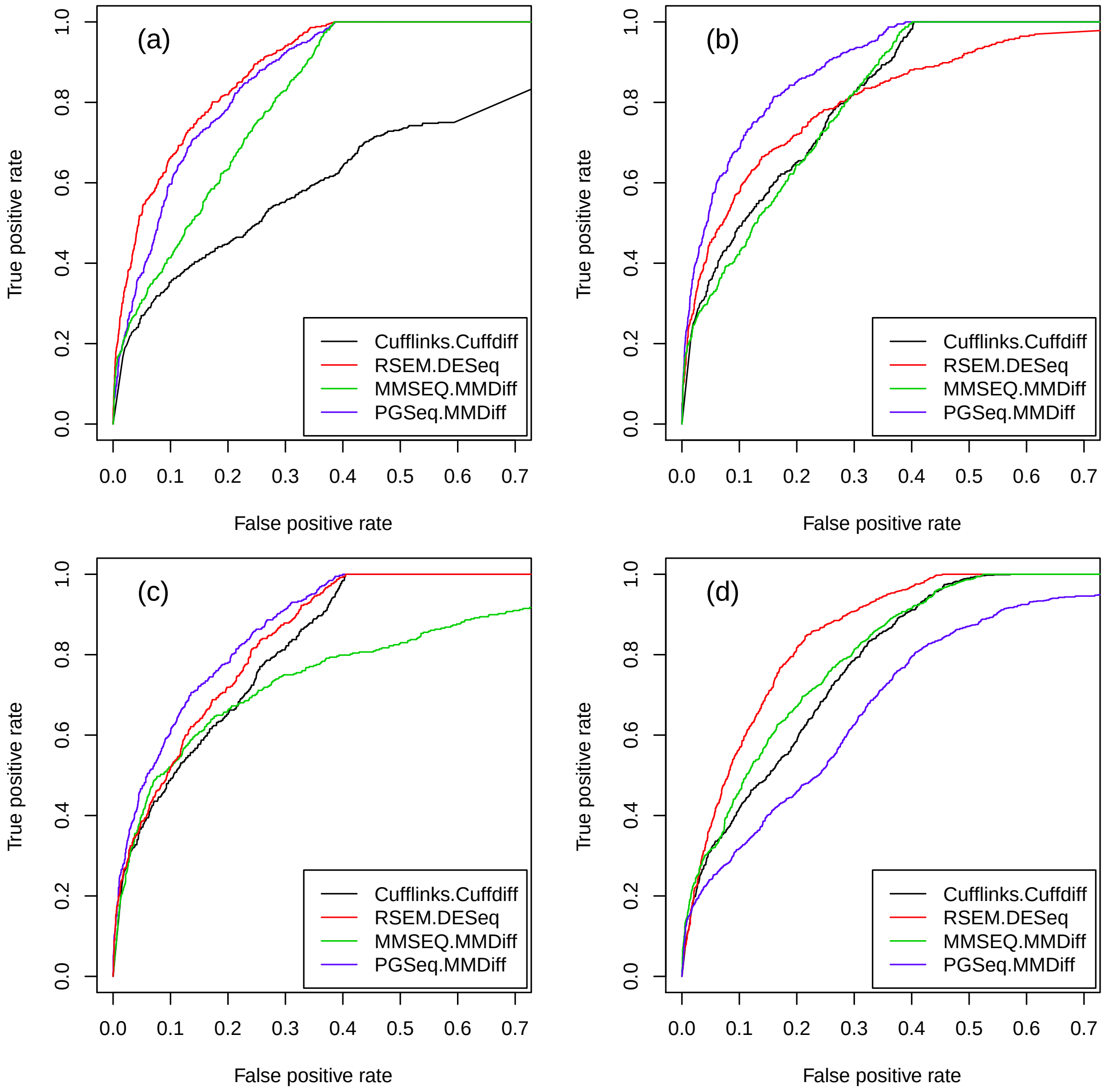

S1 Fig.: ROC curves from the four approaches for datasets validated by three methods except (a) Cufflinks, (b) RSEM, (c) MMSEQ and (d) PGSeq.
