# Supplementary material for: Modeling Exon-Specific Bias Distribution Improves the Analysis of RNA-Seq Data
Source: PLoS One. 2015 Oct 8;10(10):e0140032. doi: 10.1371/journal.pone.0140032 (PMC4598124; doi:10.1371/journal.pone.0140032)
Supplement: S2 Fig — The common DE genes (represented by red triangles) are agreed by three methods except (a) Cufflinks, (b) RSEM, (c) MMSEQ and (d) PGSeq, respectively. Others are represented by green crosses. (PDF) [file pone.0140032.s002.pdf]

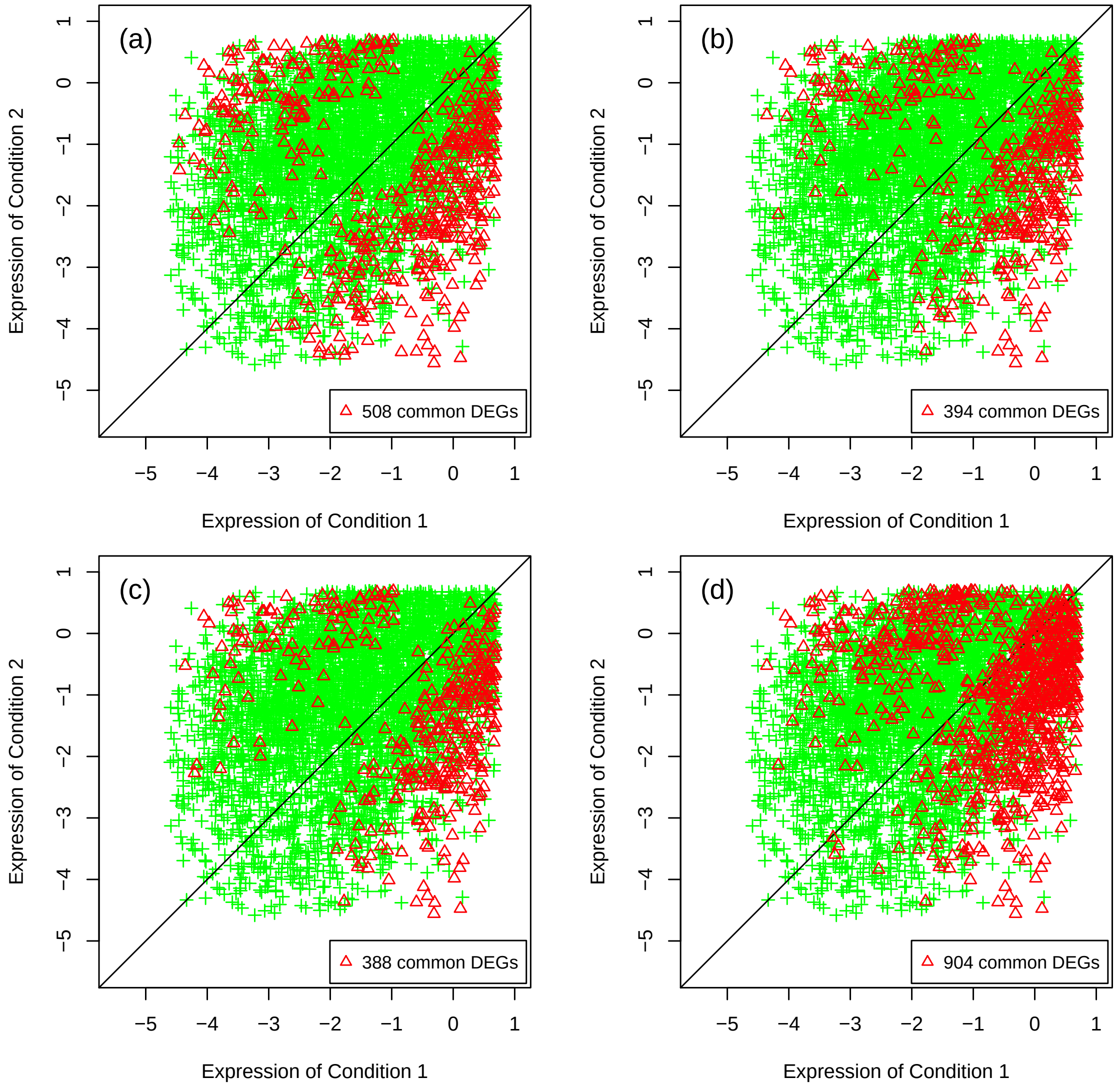

S2 Fig.: Scatter plots of the averaged logged RPKM estimation for lowly expressed genes in HDB dataset. The common DE genes (represented by red triangles) are agreed by three methods except (a) Cufflinks, (b) RSEM, (c) MMSEQ and (d) PGSeq, respectively. Others are represented by green crosses.
